# Researches on Scrofulous Diseases

**Published:** 1845-06

**Authors:** 


*Researches on Scrofulous Diseases.* By J. G. A. Lugol, D. M. P., Physician to the Hospital St. Louis, Chevalier of the Legion of Honor, &c. &c. Translated from the French. By A. Sidney Doane, A. M., M. D., late Health Officer of the Port of New York, Translator of Meckel's Anatomy, Bayle's Descriptive Anatomy, Blandin's Topographical Anatomy, Dupuytren's Surgery, Maygrier's Midwifery, Scoutetten on Cholera, Ricord on Syphilis, Chaussier on the Arteries, &c., and Editor of Good's Study of Medicine, Surgery Illustrated, &c. &c. &c. With an appendix, comprising formulæ for the the treatment of scrofula. 12mo., pp. 276. New York, 1845.

We give the whole—not simply *long* but *lengthy*—title of this small volume, inasmuch as an editor who thinks it importtant to direct attention to all the works of which he has been editor or translator, cannot fail to be gratified to see them cited by his bibliographer: at the same time, we cannot avoid expressing our doubts as to the good taste of such an ostentatious display. We know it is the custom in Europe to append to a writer's name the Societies from which he may have received valued honors; but even this is not the practice in this country: still less customary is it here, and we may add every where, for an author to enumerate on a title page a list of his other productions; and even were it a custom, we should certainly regard it as more honoured in the breach than in the observance. Dr. Doane is a most indefatigable translator, almost worthy to be classed with Jourdan, of Paris,—the great translator of medical works from the German into the French,—but who has never, we believe, published anything of his own.

The author of the work before us is well known as the urgent recommender of iodine in scrofulous diseases: his present inquiry takes for its basis, not iodine, but scrofula, which he properly regards as not merely a disease of the lymphatic ganglions, but as implicating the whole system. Strange that it should ever have been regarded otherwise!

"Scrofula," he remarks, "manifests its terrible effects in the early months of fetal existence, for it causes those spontaneous abortions which destroy at least one quarter of those affected, before they see light; after birth, it arrests their physical and moral development; it complicates all the diseases, all the evolutions of infancy and youth, which it renders laborious and full of dangers. Finally, it reveals its presence more formally by a great number of morbid states, the common origin of which has hitherto been overlooked, and which, for this reason, authors have described as so many special diseases.

Sometimes scrofula affects the mucous membranes particularly, rendering several of them diseased, and sometimes it extends its effects to the mucous system generally. Hence arise ophthalmia, coryza, otitis, leucorrhœa, intestinal worms, mucous fevers, &c.

" Sometimes it attacks the skin, and causes chilblains in the hands, feet, and face; chronic eczema of the lips, eyelids, and ears; pustules of acne scattered over the face, forehead, and chest, and pustules of lupus in various forms, in one or more regions of the dermoid system; more or less numerous, and more or less extensive ulcers, &c. It is the source of pedicular diseases, which, like the verminous affections of the intestinal canal, constantly reappear till the constitution is regenerated.

" Sometimes it acts more particularly on the cellular tissue, and produces numerous abscesses and profuse suppurations.

" When it fixes its seat specially in the osseous system, caries, necrosis, rachitism, supervene in every degree: these different alterations are rarely concentrated on one bone; most generally the bones are affected successively, and many of them are often diseased at the same time, and not unfrequently all parts of the skeleton present manifestly the impression of scrofula; but whatever may be the number of bones diseased, and the degree of the affection, they all have the same nature.

Not only are the scrofulous affections of the bones very similar to each other, but they are of the same character as the ophthalmias, otitis, &c.; as the cutaneous ulcers, and pustules, and the indolent abscesses, however numerous they are in the economy; the only difference is in the situation; this is so true that they alternate with and succeed each other, and sometimes even exist simultaneously in the same patient.

" There is not, strictly speaking, scrofula of any tissue, of any organ; it is, in all cases, the same disease, which affects in one patient the mucous system more particularly, in another the dermoid, cellular, osseous, &c., but never attacks any one of them singly. The appearance of scrofula in one organ in particular intimates its more or less proximate development in another point of the economy; often even one perceives then it had existed under other forms, which had not yet been detected."

With M. Lugol, consequently, as with MM. Rilliet and Barthez, and other recent writers, there is no difference between scrofulosis and tuberculosis.

Not fewer than one hundred and sixty pages of his book are devoted by M. Lugol to the consideration "of the inheritance of scrofulous diseases, and of the health of parents who beget scrofulous children." This comprises the first part. The second part inquires into the pathological causes; and the third part is on the external causes of Scrofula. To the whole is added an appendix, embracing formulæ for the treatment. The work is interesting as containing the views of one who has seen much of a disease which is full of interest, and at the same time of obscurity; but to the inquiring and well informed practitioner, it does not furnish much—if any—new information. Still it was well, perhaps, that it should receive an English version. It is, we think, one of the least useful of the translations executed by Dr. Doane, and such we apprehend its sale will exhibit. The translator, so far as we have observed, appears to have generally done his duty. We would suggest, however, that "ancestral parents" is not only an incorrect translation, but devoid of meaning. "Ancestral relatives,"—as he has rendered the French expression elsewhere, is the true version. Some of the formulæ, too, are inaccurate: in that for the compound tincture of iodine, *iodide of potassa* is written instead of *iodide of potassium.* The same is the case in that for the unguentum iodinii comp., which is inaccurately copied from the Pharmacopœia of London of 1836, as well as in one other. There is a manifest objection too, to the salt being termed in one prescription *iodide of potassium*, and in another *hydriodate of potassa*. It is true the adept knows that they are the same; but with the many who are not adepts confusion is inevitable.

